# Choline Analogues in Malaria Chemotherapy

**DOI:** 10.2174/138161212801327338

**Published:** 2012-08

**Authors:** Suzanne Peyrottes, Sergio Caldarelli, Sharon Wein, Christian Périgaud, Alain Pellet, Henri Vial

**Affiliations:** a Institut des Biomolécules Max Mousseron (IBMM), UMR 5247 CNRS-UM1-UM2, Université Montpellier 2, place E. Bataillon, 34095 Montpellier, France; b Dynamique des Intéractions Membranaires Normales et Pathologiques (DIMNP), UMR 5235 CNRS-UM2, Université Montpellier 2, place E. Bataillon, 34095 Montpellier, France; c Sanofi-Aventis, Research & Development, 195 route d’Espagne, BP 13669, 31036 Toulouse Cedex 1, France

**Keywords:** Malaria, plasmodium, phospholipid, choline analogues, quaternary ammonium, thiazolium salts, pharmacology.

## Abstract

Emerging resistance against well-established anti-malaria drugs warrants the introduction of new therapeutic agents with original mechanisms of action. Inhibition of membrane-based phospholipid biosynthesis, which is crucial for the parasite, has thus been proposed as a novel and promising therapeutic strategy. This review compiles literature concerning the design and study of choline analogues and related cation derivatives as potential anti-malarials. It covers advances achieved over the last two decades and describes: the concept validation, the design and selection of a clinical candidate (Albitiazolium), back-up derivatives while also providing insight into the development of prodrug approaches.

## 1 INTRODUCTION

**Fig. (1):**
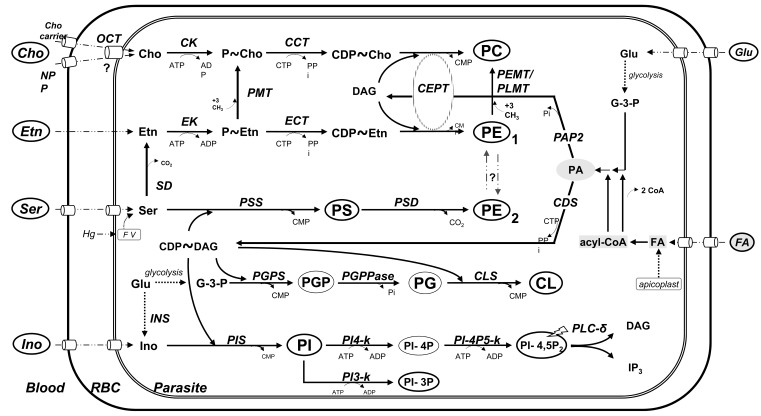
Schematic representation of phospholipid metabolic pathways in *P. falciparum* See [9] for details. Metabolites: **CL**, cardiolipin; **DAG**, diacylglycerol; **CDP**~, cytidine-diphospho-; **FA**, fatty acids; **Glu**, glucose; **G-3-P**, glycerol 3-phosphate; **Ino**, myoinositol ; **P**~, phospho; **PA**, phosphatidic acid; **PC**, phosphatidylcholine; **PE**, phosphatidylethanolamine; **PG**, phosphatidylglycerol; **PGP**, phosphatidylglycerolphosphate; **PI**, phosphatidylinositol; **PI-3P**, PI 3-phosphate; **PI-4P**, PI 4-phosphate; **PI-4-5P_2_**, PI 4,5-bisphosphate; **PS**, phosphatidylserine. Enzymes: **CDS**, Cytidine diphosphate-DAG synthase; **CK**, Cho kinase; **CCT**, CTP:P~Cho cytidylyltransferase; **CLS**, CL Synthase; **EK**, Etn kinase; **ECT**, CTP:P~#x007E;Etn cytidylyltransferase; **CEPT**, Cho/Etn-phosphotransferase; **INS**, Ino 1-phosphate synthase; **PAP2**, PA phosphatase; **PGPS**, PGP synthase ; **PGPPase**, PGP phosphatase ; **PEMT**, PE *N*-methyltransferase; **PIS**, PI synthase; **PI3-k**, PI 3-kinase; **PI4-k**, PI 4-kinase; **PI-4P5-k**, PI 4-P 5-kinase; PLC–δ, phospholipase C_δ_; **PLMT**, phosphatidyl-*N*-methylethanolamine *N*-methyltransferase; **PMME**, phosphatidyl-N-methylethanolamine; **PMT**, P~Etn Nmethyltransferase; **PSD**, PS decarboxylase; **PSS**, PS synthase CDP-DAG dependent; **SD**, Ser decarboxylase.

With an estimated 250 million infected people worldwide, malaria is considered to be one of the most lethal human diseases. The majority of these cases concern Africans, including children under 5 years old, and pregnant women are most at risk in terms of both morbidity and mortality [1-2]. Prevention and treatment currently involves both vector control, using a physical barrier between the human host and mosquitoes, and chemotherapy. However, due to the presence of four species of *Plasmodium* parasites, their geographical distribution, and the potential for the development of resistance to marketed antimalarial drugs, new treatment strategies are required [3-4]. The potential emergence of resistance to artemisinin is one of the major threats, thus highlighting the need for new chemotherapeutic approaches with novel mechanisms of action to treat *P. falciparum* infections [5-6]. Vial *et al*. [7-8] developed a therapeutic strategy based on: i) the observation that, to be able to proliferate within human erythrocytes, the parasite must produce a high quantity of phospholipids (PL) to build its membranes, which are essentially composed of phosphatidylcholine (PC) and phosphatidylethanolamine (PE), and ii) the identification of parasite metabolic pathways involved in this PL biosynthesis [9-10]. Whereas the parasite possesses its own enzymatic machinery to synthesize the required PL (Fig. **1**), it relies on its host content for starting materials, including choline (mainly), ethanolamine, serine and fatty acids. It was also demonstrated that choline transport into the infected erythrocyte is one of the limiting steps in *de novo *PC biosynthesis [11]. Interfering with this crucial step through the use of choline analogues could thus lead to a novel and promising pharmaceutical approach for malaria treatment.

## 2 FROM PRIMARY AMINES TO QUATERNARY AMMONIUM DERIVATIVES: VALIDATION OF THE APPROACH

**Fig. (2):**
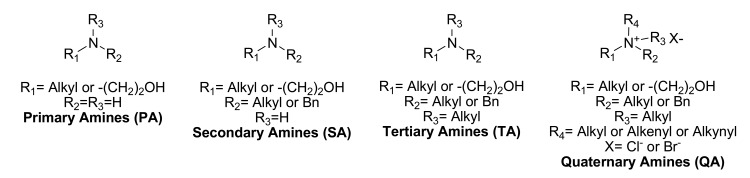
Generic structures of the studied derivatives (PA, SA, TA & QA).

The overall project started 15 years ago with the building and study of a library of choline and ethanolamine analogues (some compounds were commercially available while others were obtained in house) [12-13]. Determination of the *in vitro* antiplasmodial activity against *P. falciparum* led to the establishment of structure-activity-relationships (SARs) thus facilitating the description of essential features concerning molecular requirements to optimize our derivatives and improve their antiplasmodial potency. The screened library (about a hundred compounds) contained primary (PA), secondary (SA) and tertiary (TA) amines, and quaternary ammonium (QA) derivatives (Fig. **2**). Most of the primary amines tested were commercially available. Secondary and tertiary amines, and quaternary ammonium derivatives were obtained in high yields by alkylation of primary, secondary, or tertiary amines using the desired alkyl halide in a polar solvent [12].

All choline and ethanolamine analogues were tested *in vitro* against the growth of *P. falciparum*, the most virulent human parasite**(Tables **1** & **2**). Compounds belonging to the family of primary, secondary and tertiary amines (Table **1**, except those including a long alkyl chain, see TA8) exhibited far lower antiplasmodial activities (IC_50_ in the millimolar and micromolar range) than those containing a quaternary ammonium group (Table **2**), with 50% inhibitory concentration (IC_50_) ranging from 10^-4^ to 10^-8^ M. This first observation revealed that the positive charge is crucial for antiplasmodial activity.

Within quaternary ammonium derivatives, the effect of increasing the length of the alkyl chain from 2 to 18 carbon atoms was studied in two series, i.e. containing an hydroxyethyl moiety as in choline or not. Interestingly, the best antiplasmodial activity was observed with an alkyl chain of 12 methylene groups (IC_50_ below 1 µM) and further addition of methylene groups did not lead to a significant improvement in the IC_50_ value (Table **2**, comparison of QA6 with QA7-9). Thus, a long alkyl chain appeared crucial for antiplasmodial activity in the sub-micromolar range and the presence of an aromatic ring in the vicinity of the ammonium head did not markedly influence this activity. This latter observation shows that the targeted site is able to accommodate non-flexible substituents, but the affinity is not improved by using aromatic residues able to generate Π-Π interactions instead of lipophilic ones (i.e. in the presence of simple alkyl chains).

Besides this, for compounds with the same alkyl chain length, the presence of an hydroxyethyl group on the nitrogen atom was not essential for high antiplasmodial activity. 

For active compounds of the QA series, we also decided to bulk the substituents around the nitrogen atom. Replacement of the three methyl substituents by ethyl or propyl residues thus significantly improved the activity, indicating that an increase in steric hindrance and/or lipophilicity around the nitrogen atom is beneficial for antiplasmodial potency. In the choline analogue series, *N*,*N*-diethyl substitution did not modify the efficacy. Moreover, the presence of a second long alkyl chain (12 carbon atoms) grafted to the nitrogen atom did not improve the IC_50_ compared to a methyl substituent, and in some cases was even unfavorable.

SAR analyses have shown that *in vitro* antiplasmodial activities against the human parasite *P. falciparum* were related to the shape, electronegativity and lipophilicity of the compound. A positive charge located on the nitrogen and adjustments of shape and size appear to be crucial features for the optimization of these competitive reversible inhibitors. The targeted site likely contains an anionic or a high electron density region able to accept a polar and cationic head (*N*,*N*,*N*-trimethyl, *N*,*N*-dimethyl-*N*-hydroxyethyl, or *N*,*N*-diethyl-*N*-hydroxyethyl). Analogues bulkier than choline (*N*,*N*,*N*-triethyl, *N*,*N*,*N*-tripropyl groups) likely highly interfere with the binding site, however compounds including a second long alkyl chain (C12) appeared too large and are consequently less potent. Clearly, this highlighted that the nature of substituents around the nitrogen atom should be carefully chosen and are essential for optimal interactions. We also hypothesized that a large and non-polar region is located close to the polar head binding site and is able to accept only one long alkyl chain.

In summary, the most potent *in vitro *activities were obtained for *N*-dodecyl-substituted ammonium derivatives.

## 3 BIS(QUATERNARY AMMONIUM) DERIVATIVES: PHARMACOPHORE ESTABLISHMENT

**Scheme 1.**
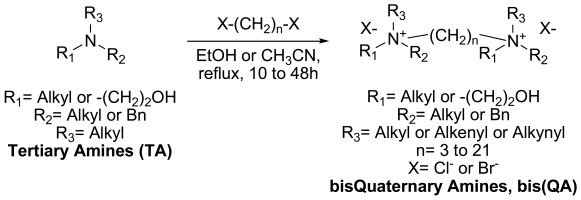
Generic structures and synthetic approach of the studied bis(QA) derivatives.

With the aim of improving the antiplasmodial activity of the previous quaternary ammonium derivatives, a new series of compounds was envisaged by designing duplicated molecules incorporating two polar heads linked by an alkyl chain. Indeed, by combining two pharmocophores as parts of a single molecule, one could expect to obtain a more effective drug that should be capable of interacting simultaneously with more than one targeted site. Furthermore, the chemical bridge (linker in between the two polar heads) itself might also participate in the biological effect. Therefore, symmetrical bis quaternary ammonium salts with various lipophilic substituents on the nitrogen atom and different alkyl chains lengths (3 to 21 methylene groups) were synthesized (Scheme **1**) and then studied for their antimalarial potential [12, 15]. 

We were pleased to observe that the studied derivatives exhibited a wide range of antiplasmodial activities (Table **3**), with IC_50_ values ranging from 10^-4^ M to 10^-12^ M, i.e. up to 4 orders of magnitude lower than mono quaternary ammonium salts (see above). As previously, we investigated the effects of various modifications on *in vitro* antiplasmodial activity, such as lipophilicity by increasing the inter-nitrogen chain length or by adding electronegative or electron-rich *N*-substitutions.

Thus, the structural requirements for antiplasmodial activity of bis(QA) salts were very similar to those of mono(QA) salts, i.e. polar head steric hindrance and lipophilicity around nitrogen (methyl, hydroxyethyl, ethyl, pyrrolidinium, etc…). Within the bis(QA) series, increasing the lipophilicity of the alkyl chain between the two nitrogen atoms (from 5 to 21 methylene groups) constantly improved the activity. Most of these duplicated molecules exhibited activity within the low or sub-nanomolar ranges, and the most lipophilic compound had an IC_50_ as low as 3 pM (Table **3**, n=21 methylene groups, compound G19). As the polymethylene chain linking the positively charged heads should contain more than 10 methylene groups to obtain bis(QA) derivatives substantially more potent than their corresponding mono(QA)salts, it was suggested that the two targeted sites may be vicinal and separated by an hydrophobic domain. 

Concerning modification of the bulk of the cationic head, a significant difference was observed for *N*-methylpyrrolidinium derivatives in comparison to their corresponding trimethyl and triethyl analogues (Table **3**, see derivative named G25). This indicates that the pocket is large enough to accommodate such a large head.

**Fig. (3):**
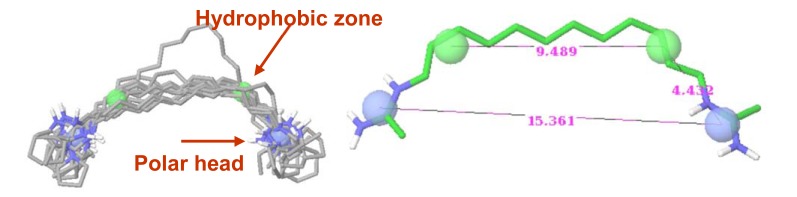
Proposed pharmacophore description (M. Bianciotto, published with permission).

On the basis of the overall data for both mono(QA) and bis(QA) salts, we estimated that the site targeted by the cationic head may be viewed as a globular volume of 200 to 350 Å^3^, which corresponds to a radius of ~4 Å. Our results were also consistent with the targeting of two vicinal sites. With alkyl chains longer than 14 methylene groups (20 Å), the distance between the two cationic heads appeared to be optimal for the concomitant interaction with two binding sites of the target (Fig. **3**). 

To the best of our knowledge, this new family of derivatives appears to be one of the most potent classes of antimalarials in the development stage [16-18]. Among currently marketed antimalarials, only halofantrine [19] and artemisinin [20] analogues have exhibited IC_50_ values in the low nanomolar range. 

Further *in vitro* biological studies [21-22] were performed and some selected derivatives were tested against various chemoresistant *P. falciparum *strains or isolates, and they showed the same high activity as against sensitive strains. More specifically, the G25 compound was equally active against the two chloroquine-resistant strains FCR3 and L1 (chloroquine IC_50_ 0.8 and 0.9 µM, respectively) with an IC_50_ of 3.6 and 1.4 nM, respectively, while also exhibiting potency (nanomolar range) against various human isolates (such as cycloguanil-, chloroquine-, or mefloquine-resistant isolates).

On the basis of these encouraging results, we then focused on determining the mechanism of action of such derivatives, which were designed as choline analogues in order to interfere with the parasite’s phospholipid biosynthesis. In this respect, the relationship between the *in vitro* *P. falciparum *growth and the inhibition of the phospholipid metabolism was studied as well as the effect of the compounds on choline transport [21, 23]. One prominent feature of the biscationic analog G25 is its ability to accumulate, by several hundredfold, within *P. falciparum*-infected erythrocytes, which is already significant at the ring stage and increased as the parasite matures. This nonreversible accumulation is likely important with respect to the mechanism of action and appears to determine its specificity and potency [24].

**Fig. (4):**
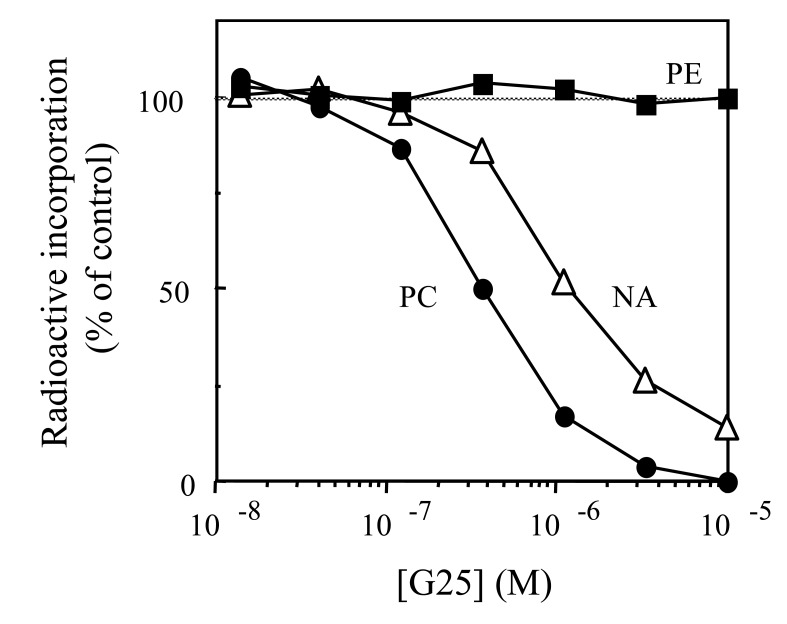
Effect of G25 on [^3^H]choline, [^3^H]ethanolamine and [^3^H] hypoxanthine incorporation into PC, PE and nucleic acids (NA) of *P. falciparum*-infected erythrocytes. Infected red blood cells (RBC, 3.4% hematocrit, 10% parasitemia, Nigerian strain) were incubated for 4 h at 37°C in the absence or presence of the studied compound (G25) as well as in the presence of 1.5 µCi of [^3^H]choline (●, at 10 µM), 0.8 µCi of [^3^H]ethanolamine (■ at 2 µM) or 1 µCi of [^3^H] hypoxanthine (Δ, trace amounts).

For all 50 derivatives studied, a close correlation between the PL metabolism impairment and the parasite growth inhibition was observed. Some evidence that these compounds act by PL metabolism inhibition was also obtained when an excess of choline was used, which led to reversal of the PC metabolism inhibition. As shown in (Fig. **4**), the typical dose-response curves obtained with one bis(QA) salt, namely G25, confirmed that specific inhibition of choline incorporation into PC (PC_50_ 0.4 µM, 4 h incubation) had occurred, whereas inhibition of nucleic acid and PE biosynthesis were observed only at higher concentrations. Furthermore, for the bis(QA) salts, the PC_50_s were 3 to 40 times lower than the NA_50_, indicating specificity with respect to *de novo* PC biosynthesis. 

Thus, a positive relationship was established between the ability of the drugs to inhibit parasite growth in culture and their capacity to specifically inhibit phosphatidylcholine biosynthesis of *P. falciparum- *infected erythrocytes.

The effect of G25 on choline transport was also assessed [21-22] in *P. knowlesi*-infected RBC and in *Saccharomyces cerivisiae* cells. The inhibition pattern was sigmoidal, occurring over 1 to 2 orders of magnitude. The G25 concentration leading to 50% inhibition of choline influx was 0.8 µM, which corresponds (assuming that inhibition was competitive) to a calculated *Ki *of 0.4 µM [25], which is exactly the PC_50_ value. Pretreatment of healthy RBC with parasite-lethal concentrations of G25 did not change the susceptibility of RBC to *P. falciparum* invasion and their ability to support parasite growth, indicating that the G25 toxic effect did not occur at the host cell level. The time course of *in vitro* *P*. *falciparum *growth inhibition as a function of parasite development was also determined. For the G25 model compound, the best activity was observed for the trophozoite stage in the 48 h erythrocytic cycle (IC_50_ 0.75 nM), whereas the schizont and ring stages were 12- and 213-fold less susceptible. Of upmost importance, the compounds exerted a rapid nonreversible cytotoxic effect with complete clearance of parasitemia after 5 h of contact with the mature stages.

In the meantime, *in vivo* experiments were carried out [26] in several murine malarial models to demonstrate the full potential of this family of derivatives (Table **4**). Tolerance after administration to mice was first investigated after intraperitoneal (i.p.) administration, and the acute 50% lethal dose (LD_50_) generally ranged from 0.15 to 50 mg/kg. Toxicity increased with steric hindrance around the nitrogen molecule or lengthened the alkyl chain from 12, 16 to 21 methylene groups.

Three distinct murine models were used to determine *in vivo* antimalarial activities, i.e. *P*. *berghei*-, *P*. *chabaudi*-, or *P. vinckei*-infected mice. The two latter murine strains differ from the former by their marked preference for invading mature erythrocytes and their high degrees of synchronization [27-28]. Most of the tested compounds were able to clear parasitemia, which is evidence of their antimalarial potencies. Against *P. berghei*, the ED_50_ after i.p. administration (once-daily for 4 days) ranged from 0.04 to 0.3 mg/kg for bis-QA compounds, and even lower against *P*. *chabaudi*. 

The *in vivo* selectivity (therapeutic index, TI) increased slightly as the polar head volume decreased. Similar TI values were observed for derivative G25 when the *in vivo* antimalarial activity was investigated after i.p., s.c., or oral administration. However, there was a 90-fold difference in LD_50_ (or ED_50_) between the i.p. and oral modes, which indicates that the oral absorption of this compound is very low. In fact, oral uptake for such bis cationic derivatives was expected to be weak due to charges.

In conclusion, PL metabolism of *P falciparum*-infected erythrocytes, especially *de novo* PC biosynthesis from choline, is thus a quite realistic target for our derivatives. The efficacies of bis(QA) salts appeared to be consistent with the presence of two anionic sites on the choline carrier separated by a large lipophilic domain. This impressive and selective *in vitro* toxicity against *P. falciparum, *the high therapeutic index observed in infected mouse models and the absence of recrudescence strongly highlights the clinical potential of these bis(quaternary ammonium) salts for malarial chemotherapy.

## 4 MONO- AND BIS(THIAZOLIUM) SALT STUDY: SELECTION OF ALBITIAZOLIUM AS CLINICAL CANDIDATE

**Fig. (5):**
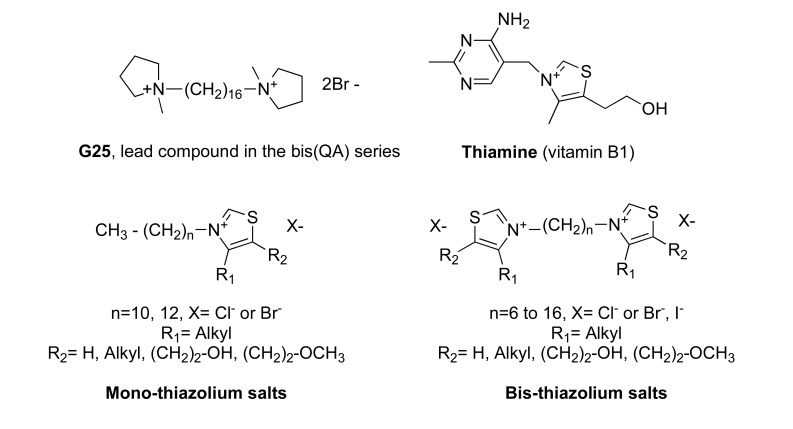
Generic structures of the studied mono- and bis(thiazolium) derivatives, vitamin B1 and lead compound G25 in the bis(QA) series.

In the bis(QA) series, the G25 derivative (Fig. **5**) appeared to be one of the most active compounds against *P. falciparum *(IC_50_ 0.65 nM) and was able to cure, without recrudescence, *P. falciparum*-infected *Aotus *monkeys at the very low dose of 0.03 mg/kg [24]. However, this is hampered by the very limited oral bioavailability and toxicity at doses higher than 1.5 mg/kg due to the effect on the cholinergic system (H. Vial, unpublished data). To overcome this problem, we envisaged the replacement of the quaternary ammonium group by a thiazolium ring. This heterocycle presents two main advantages, it is much less toxic and present in a naturally occurring thiazolium derivative, i.e. vitamin B1, and allows the design of neutral prodrugs to improve their bioavailability, as already described for vitamin B1 [29-31].

As a follow-up to our previous studies on mono- and bis(QA) compounds, we built-up and studied an oriented library of mono- and bis cationic derivatives incorporating thiazolium moiety(ies). All the synthesized derivatives were evaluated for their *in vitro* antiplasmodial activity on *P. falciparum*. Among the most active compounds (IC_50_ in the low namolar range), several were tested for *in vivo* inhibition of *P. vinckei *growth in mice (Table **5**) [32]. 

The conclusions of the structure-activity relationship (SAR) study were:

- As for QA-based compounds, duplication of the polar head, when using a dodecyl chain, led to an impressive improvement, i.e. by 2- to 3-log scales of the antiplasmodial activity in comparison to the corresponding monothiazolium derivatives
- the length of the alkyl chain (from C8 to C16) is a crucial element for high antiplasmodial activity. It indicate an optimum for 12 methylene groups (as compared to more than 16 observed for bis(QA))
- the nature of the R_2_ substituent at the C-5 position of the thiazolium ring (Fig. **5**) also has a strong influence. Briefly, molecules including a small size group (R_2_ = Me, Et, (CH_2_)_2_OH, (CH_2_)_2_OMe) were more active than those unsubstituted (R_2_ = H) or bearing a bulky substituent [R_2_ = (CH_2_)_2_OiPr, (CH_2_)_2_Cl, (CH_2_)_2_OCO(CH_2_)_2_CO_2_Me]. The optimal substituent at the C-5 position was a methoxyethyl group.

The two most potent compounds (named by us **T3/SAR97276 **and **T4,** respectively) were selected for further development on the basis of their *in vivo* activity by the i.p. route against *P. vinckei *in**infected mice (ED_50_ 0.2 and 0.14 mg/kg, respectively), their superiority to chloroquine (ED_50_ 1.1 mg/kg), and their good tolerance (therapeutic index > 50). These compounds were thus evaluated for their pharmacological potency in very severe conditions such as high parasitemia or in short course treatments with a single injection [33]. After a daily administration of T3/SAR97276 for 4 days, mice were completely cured, with an ED_50_ below 0.3 mg/kg using ip, im or iv routes. Remarkably, the ED_50_ of T3/SAR97276 was in the same range at low and high initial parasitemia: 0.3 mg/kg and ≤ 0.5 mg/kg, respectively. ED_90_ and complete cure without recrudescence were obtained at 2- to 4-times the ED_50_. A single injection of T3/SAR97276 at a dosage of 6.75 mg/kg led to a complete cure of *P. vinckei*-infected mice infected at low or moderate parasitemia, thus highlighting the potency of the compound.

T3/SAR97276 was also evaluated in comparison to artesunate, whose remarkable antimalarial activity is associated with a high parasite killing rate [34]. Our lead compound exhibited higher activity by the i.p. route (ED_50_ = 0.5 mg/kg) than artesunate (ED_50_ >2.5 mg/kg). Moreover, a total cure was obtained with T3/SAR97276 at a daily dose of 0.5 mg/kg for 4 days, this was not obtained with an artesunate dosage of even 10 mg/kg.

In addition, the bisthiazolium salts appeared to be specific inhibitors of malarial phosphatidylcholine biosynthesis [33] and also accumulated to high extent in hematozoan-infected erythrocytes (*Plasmodium* and *Babesia*) [35-36]. A substantial share of the accumulated drugs appeared to be localized within the parasite’s digestive vacuole and their interaction with heme, the non-protein part of haemoglobin, was suggested to contribute to their potent antimalarial activity [36]. 

In the light of the overall pharmacological, biological, ADME and toxicological data generated in collaboration with an industrial partner (unpublished), the findings of solubility and formula studies, and the low cost and ease of synthesis, the bis-thiazolium salt T3/SAR97276 was selected as a clinical candidate. Regulatory preclinical and Phase I clinical studies were successfully carried out by Sanofi-Aventis. In 2008, phase II studies were initiated for the treatment of severe *P. falciparum* malaria *via* the parenteral route in several research centers in Africa. This open-label, nonrandomized, non-comparative Phase II study was aimed at assessing the antimalarial activity, safety, and pharmacokinetic profile of T3/SAR97276, renamed Albitiazolium, following single and repeated administrations *via* intravenous (i.v.) and intramuscular (i.m.) routes. 

Meanwhile, we have a global research plan to obtain an orally available formulation of T3/SAR97276 and/or a related analogue that could be developed for the treatment of uncomplicated malaria. Indeed, even though bis(thiazolium) salts have already been shown to achieve a complete cure after oral administration, their intestinal absorption appears too low to warrant pharmaceutical development and this aspect needs to be optimized. In this respect, two approaches have been developed in parallel (see below): the first one is based on the design of neutral prodrugs or bioprecursors of the parent drugs, while the second deals with the synthesis and study of Albitiazolium analogues with more favorable molecular parameters (i.e. molecular weight, flexibility and lipophilicity). 

## 5 EXPLORING PRODRUG APPROACHES FOR ORAL DELIVERY

**Scheme 4.**
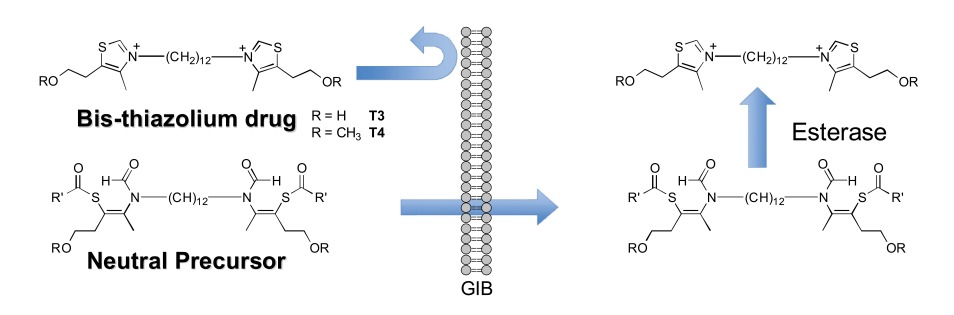
Neutral prodrug approach to optimize the oral bioavailability of bis-thiazolium **T3** and **4**

The derivatives described above exhibit a low oral bioavailability mainly due to the presence of permanent cationic charges, which is crucial for antiplasmodial activity. This prompted us to develop neutral precursors of the corresponding bis-thiazolium salts (Scheme **4**), which should be quantitatively and rapidly converted *in vivo* to the active parent drug after crossing the gastrointestinal barrier [33, 37]. The prodrugs studied thus incorporated thioester, thiocarbonate or thiocarbamate pro-moieties, which are expected to be hydrolyzed *in vivo* to the parent drugs after enzymatic transformation involving plasmatic esterases. 

The synthetic pathways developed take advantage of the particular reactivity of the thiazolium ring which can be opened in basic aqueous conditions [37]. Thioester, thiocarbonate or thiocarbamate derivatives were thus obtained in one step by reaction of an alkaline solution of the parent drug (T3 or T4) with the appropriate activated acyl group. A series of 37 neutral precursors was obtained using a low cost synthetic pathway, with yields ranging from 15% to 96%, depending on the nature of the reagents used (i.e. acyl- and arylchloride, alkyl chloroformate or activated carboxylic acid, etc.). Structural variations (lipophilicity, steric hindrance, electronic effect, etc.) affecting the physicochemical properties were made in order to study their influence on oral activity.

All prodrugs were evaluated for their antimalarial activity *in vitro* against *P.* *falciparum* and *in vivo* against *P. vinckei* (Tables **6** & **7**). Twenty-five of them exhibited potent *in vitro* antiplasmodial activity, with an IC_50_ below 10 nM, indicating quantitative conversion of the prodrugs into their parent drugs. Furthermore, four derivatives were as potent as the parent drugs T3 and T4, by either i.p. and *per os* (p.o) administration. 

In the thioester series (Table **6**), the steric hindrance (Me, Et, iPr, tBu, etc.) and/or lipophilicity of the alkyl residue demonstrated a modest effect on antimalarial activity. Introduction of a phenyl group was tolerated, but the presence of hindered substituents on the aryl dramatically increased the IC_50_ (from nM to µM range) and this lack of *in vitro* activity was attributed to a slower prodrug-drug conversion rate. Significant differences were observed when decreasing the pro-moiety lipophilicity by introducing polar functional groups (ether, ketone or ester). Notably, when R= (CH_2_)_2 _COMe, the prodrug exhibited an IC_50_ of 2.2 nM and an ED_50_ of 3 mg/kg *per os*.

Within thiocarbonate derivatives (Table **6**), the minimal substituent (methoxy group) appeared to be the best one, with an IC_50_ of 1.8 nM, and this compound was the most powerful after oral administration, with an ED_50_ of 1.3 mg/kg.

When using T3 as parent drug (Table **6**), only thioester prodrugs were available due to synthetic problems. Thus similar effects of the nature of the acyl substituents upon biological activity were observed as for the corresponding T4 prodrugs. More interestingly, to generate low molecular weight prodrugs the cyclic thiocarbonate and dithiocarbonate (that should be more stable towards esterase hydrolysis) prodrugs were designed and prepared. Whereas the dithiocarbonate derivative was not active (IC_50_ of 4400 nM and ED_50 _ip>30 mg/kg), the cyclic thiocarbonate compound showed an *in vitro* IC_50_ = 2.2 nM and the oral activity was enhanced (ED_50 _= 5 mg/kg) in comparison to T3. Unfortunately, pharmacokinetics data showed that this compound is rapidly converted into T3 in the gastrointestinal tract. 

Structural variations of the pro-moieties were designed to affect the lipophilicity, molecular weight and enzymatic stability. These results show that the common features of the most active compounds are a low molecular weight and a low clog P compared to weaker compounds.

A new series of prodrugs are currently being developed with the aim of preventing early prodrug-drug bioconversion and/or increasing the aqueous solubility, with both parameters affecting oral absorption. A new prodrug in the thioester family has thus shown encouraging preliminary results and deserves further attention. Findings of preliminary studies on the efficacy and tolerance of these compounds in mice and in primates appear promising, indicating that this approach may be applicable to human malaria.

## 6 BACK-UP DERIVATIVES: MODIFICATIONS OF THE LINKER

Bis-thiazolium salts exhibited weak oral bioavailability (< 5%) so there was no further clinical development for oral administration. Nevertheless, such derivatives could be administered as neutral precursors that are expected *in vivo *to revert back to the parent drugs (see above). The use of a cyclic prodrug approach led to improvement of the absolute oral bioavailability of T3 (reaching 15%) but it remained weak.

Careful analysis of the previous results showed that the reduction in the overall flexibility of the molecule, in the case of neutral prodrugs, led to an increased ip/po ratio. Moreover, it is well established that physico-chemical properties (hydro solubility, flexibility, etc.) are critical parameters for designing oral-drug like compounds. Improving such properties for bis-thiazolium salts, and corresponding prodrugs, has been envisaged through modification of the linker between cationic heads which is highly flexible (11 C-C bonds), lipophilic and thus may not have favorable parameters for oral bioavailability.

**Scheme 5.**
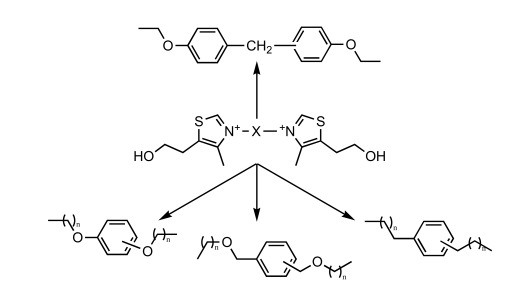
Proposed modifications in the dodecyl linker.

As an example, the aromatic ring has been introduced to decrease the flexibility, and polar atoms (oxygen ones) have been incorporated to decrease the lipophilicity of the related prodrugs (Scheme **5**) [39]. For each aromatic ring, different methylene arm lengths (n= 3, 4, 5) have been tested and modulation of the anchoring position (ortho, meta, para) has been explored.

All compounds were then evaluated on *P. falciparum*-infected erythrocytes and against *P. vinckei* in mice for their antimalarial properties (Table **8**).

The *in vitro* activity ranged from 9 nM to 800 nM. Lengthening of the linker led to an increased antiplasmodial activity and the presence of oxygen atoms was somehow detrimental to the antiplasmodial activity. *Para* and *meta* compounds exhibited lower IC_50_ values for compounds with the same chain length but anchored at different positions on the aromatic ring.

The structure-activity relationship suggested that the optimal linker-construct was an aromatic moiety branched with two *n*-butyl chains in the *para* orientation. Two promising compounds incorporating modified linkers were identified and were able to cure malarial infection in mice at very low doses (i.e. IC_50 _≤ 20 nM and ED_50_ ip < 5 mg/kg). A prodrug approach will soon be applied to evaluate the effect of the linker modifications on oral absorption enhancement.

## 7 CONCLUDING REMARKS

Phospholipids are crucial for the development of intracellular malaria parasites. Consequently, proteins involved in their biosynthetic pathways often appear essential [40-41] and thereby represent potential targets for antimalarial drugs. Our main goal is to develop new antimalarial drugs that will affect their biosynthetic pathways with an innovative mechanism of action.

Based on **i)** crucial biosynthetic pathways, **ii)** limiting steps within the metabolic pathways, and **iii**) specificity regarding the host, we have identified phospholipid-related transporters or enzymes suitable for drugability and for pharmacological targeting.

We rationally designed choline analogs and optimized them for their antiplasmodial activity. The end products are compounds able to block *P. falciparum* asexual blood stages, at single digit nanomolar concentrations, and able to cure malarial infection in rodents or non-human primates. The potency and specificity of these antiphospholipid effectors are likely due to their unique ability to accumulate in a nonreversible way inside the intraerythrocytic parasite. These compounds are thought to inhibit choline transport, thus preventing PC synthesis, and also to interact with plasmodial haemoglobin degradation metabolites in the food vacuole. This multiple mode of action, distinct from current antimalarial agents, is the major asset of this inhibitor class and this feature could also help delay the development of resistance.

We have made considerable progress from mono-quaternary ammoniums to bis-thiazolium salts in blocking the biosynthetic route of phosphatidylcholine, the major malaria lipid. One choline analog has been identified as a clinical candidate and Albitiazolium has moved progressively to phase II human clinical trials, which are under way with respect to achieving a parenteral cure for severe malaria.

The current objective is to design and select an orally available analog as a clinical candidate for the treatment of uncomplicated malaria *in *the field.

## Figures and Tables

**Table 1.** Selected *In Vitro* Antiplasmodial Activities (IC*50*) Against *P. falciparum* for the Studied Derivatives (PA, SA & TA)*

Given name	R_1_	R_2_	R_3_	IC_50_ (µM)
PA0	HO-(CH_2_)_3^-^_	H	H	800
PA1	HO-(CH_2_)_5^-^_	H	H	> 2000
PA5	(HO-CH_2_)_2_CH-	H	H	50
PA9	H_2_N-(CH_2_)_5^-^_	H	H	2000
SA1	HO-(CH_2_)_2^-^_	HO-(CH_2_)_2^-^_	H	1100
SA2	HO-(CH_2_)_2^-^_	C_6_H_5^-^_CH_2_-	H	40
**SA3**	**HO-(CH_2_)_2^-^_**	**C_12_H_25^-^_**	**H**	**0.51**
TA5	HO-(CH_2_)_2^-^_	CH_3^-^_	CH_3^-^_	1300
TA6	HO-(CH_2_)_2^-^_	C_2_H_5^-^_	C_2_H_5^-^_	400
TA7	HO-(CH_2_)_2^-^_	(CH_3_)_2_CH-	(CH_3_)2CH-	450
**TA8**	**HO-(CH_2_)_2^-^_**	**CH_3^-^_**	**C_12_H_25^-^_**	**1.3**
TA1	HO-(CH_2_)_2^-^_	-(CH_2_)_2^-^_		50
TA2	HO-(CH_2_)_2^-^_	-(CH_2_)4-		420
TA3	HO-(CH_2_)_2^-^_	-(CH_2_)_5^-^_		350

* Overall *in vitro* biological data were reported in reference [12] and measured after contact with the blood stage for one full cycle (48 h)

**Table 2.** Selected *In Vitro* Antiplasmodial Activities (IC_50_) Against *P. falciparum* for QA Derivatives*

Given name	R_1_	R_2_	R_3_	R_4_	IC_50_ (µM)
QA2	CH_3^-^_	CH_3^-^_	CH_3^-^_	C_7_H_15^-^_	300
QA4	CH_3^-^_	CH_3^-^_	CH_3^-^_	C_10_H_21^-^_	0.7
QA6	CH_3^-^_	CH_3^-^_	CH_3^-^_	**C_12_H_25^-^_**	**0.5**
QA7	CH_3^-^_	CH_3^-^_	CH_3^-^_	C_14_H_29^-^_	0.9
QA8	CH_3^-^_	CH_3^-^_	CH_3^-^_	C16H3_3^-^_	0.8
QA9	CH_3^-^_	CH_3^-^_	CH_3^-^_	C_18_H_37^-^_	2.1^a^
QA20	CH_3^-^_	CH_3^-^_	C_2_H_5^-^_	C_12_H_25^-^_	0.11
QA22	CH_3^-^_	CH_3^-^_	C_4_H_9^-^_	C_12_H_25^-^_	0.26 ^b^
QA24	CH_3^-^_	-(CH_2_)_4^-^_		C_12_H_25^-^_	0.22
QA30	CH_3^-^_	CH_3^-^_	C_12_H_25^-^_	C_12_H_25^-^_	0.7
QA40	CH_3^-^_	CH_3^-^_	C_6_H_5^-^_CH_2^-^_	C_14_H_29^-^_	1 ^a^
QA10	C_2_H_5^-^_	C_2_H_5^-^_	C_2_H_5^-^_	C_12_H_25^-^_	0.064
QA13	C_3_H_7^-^_	C_3_H_7^-^_	C_3_H_7^-^_	C_12_H_25^-^_	0.033
QAF3	HO-(CH_2_)_2^-^_	CH_3^-^_	CH_3^-^_	C_10_H_21^-^_	0.97
QAF4	HO-(CH_2_)_2^-^_	CH_3^-^_	CH_3^-^_	C_12_H_25^-^_	0.48
QAF5	HO-(CH_2_)_2^-^_	CH_3^-^_	CH_3^-^_	C_14_H_29^-^_	0.6
QAF6	HO-(CH_2_)_2^-^_	CH_3^-^_	CH_3^-^_	C_18_H_37^-^_	1.3
QAF7	HO-(CH_2_)_2^-^_	CH_3^-^_	C_12_H_25^-^_	C_12_H_25^-^_	0.84
QAF12	HO-(CH_2_)_2^-^_	CH_3^-^_	CH_3^-^_	C_6_H_5^-^_CH_2^-^_	112
QAF13	HO-(CH_2_)_2^-^_	CH_3^-^_	CH_3^-^_	C_6_H_5^-^_(CH_2_)_2^-^_	110
QAF15	HO-(CH_2_)_2^-^_	CH_3^-^_	CH_3^-^_	C_6_H_11^-^_	120

* Overall *in vitro* biological data were reported in references [12, 14]. Whereas most QA derivatives were isolated as bromine salt, compounds marked with a or b were obtained as chlorinate or iodinate salt, respectively.

**Table 3.** Selected *In Vitro* Antiplasmodial Activities (IC_50_) Against *P. falciparum* for bis(QA) Derivatives*

Given Name	R1	R2	R3	n	IC_50_ (µM)
G1	CH_3^-^_	CH_3^-^_	CH_3^-^_	6	700
G4	CH_3^-^_	CH_3^-^_	CH_3^-^_	12	0.09
G5	CH_3^-^_	CH_3^-^_	CH_3^-^_	16	0.004
G45	CH_3^-^_	CH_3^-^_	C_12_H_25^-^_	16	1.8
G24	CH_3^-^_	-(CH_2_)_4^-^_		12	0.013
**G25**	**CH_3^-^_**	**-(CH_2_)_4^-^_**		**16**	**0.00064**
G14	C_2_H_5^-^_	C_2_H_5^-^_	C_2_H_5^-^_	12	0.045
G15	C_2_H_5^-^_	C_2_H_5^-^_	C_2_H_5^-^_	16	0.0016
**G19**	**C_2_H_5^-^_**	**C_2_H_5^-^_**	**C_2_H_5^-^_**	**21**	**3 × 10^-6^**
H5	C_2_H_5^-^_	C_2_H_5^-^_	HO-(CH_2_)_2^-^_	16	0.0049

* Overall *in vitro* biological data were from references [12, 15].

**Table 4.** Selected *In Vivo* Antimalarial Activity (ED_50_), Acute Toxicity (LD_50_) and Therapeutic Index (TI) for bis(QA) Derivatives after i.p. Administration*

Given name	R_1_	R_2_	R_3_	n	IC_50_ (nM)	ED_50_ (mg/kg)	LD_50_ (mg/kg)^c^	TI^d^
G5	CH_3^-^_	CH_3^-^_	CH_3^-^_	16	4	0.11^a^	3	27^a^
						0.06^b^	-	50^b^
G24	CH_3^-^_	-(CH_2_)_4^-^_		12	13	ND	ND	ND
G25	CH_3^-^_	**-(CH_2_)_4^-^_**		**16**	**0.064**	**0.21^a^**	1,4	7 a
						**0.08 b**	-	18 b
G14	C_2_H_5^-^_	C_2_H_5^-^_	C_2_H_5^-^_	12	45	>0.4	1	>2.5
**G15**	**C_2_H_5^-^_**	**C_2_H_5^-^_**	**C_2_H_5^-^_**	**16**	**1.6**	**0.04 a**	**0.2**	**5 a**
						**0.023 b**	**-**	**9 b**

* Overall biological data were reported in reference [15, 26].

a ED_50_ were determined in a *P. berghei* NS murine model

b ED_50_ were determined in a *P. chabaudi* murine model

c LD_50_ are values obtained for *in vivo P. berghei* or *P. chabaudi* per mouse

d Therapeutic index (TI) corresponds to the LD50 ip/ED50 ip ratio.

**Table 5.** Selected In Vitro (IC50) and In Vivo Antimalarial Activities (ED50) for bis(Thiazolium) Derivatives after i.p. Administration*

Given name	R_1_	R_2_	n	IC_50_ (nM)	ED_50_ i.p. (mg/kg)
T3.8	CH_3_-	(CH_2_)_2_-OH	8	362	ND
T3.10	CH_3_-	(CH_2_)_2_-OH	10	34	ND
T4.10	CH_3_-	(CH_2_)_2_-OCH_3_	10	8.9	ND
T6	CH_3_-	H	12	3	2.6
**T3**	**CH_3_-**	**(CH_2_)_2_-OH**	**12**	**2.25**	**0.2**
**T4**	**CH_3_-**	**(CH_2_)_2_-OCH_3_**	**12**	**0.65**	**0.14**
T14	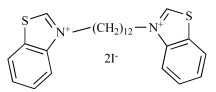			2300	ND
T2	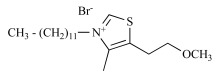			75	ND

* Overall biological data were from reference [32].

**Table 6.** Selected *In Vitro* (IC_50_, Against *P. falciparum*) and *In Vivo* (ED_50_, Against *P. vinckei*) Antimalarial Activities of Thioester, Thiocarbonate and Thiocarbamate Prodrugs and the Corresponding Drug T4*

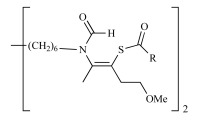	**IC_50_ (nM)**	ED_50 _(mg/kg) i.p. p.o.	
T4 (parent drug)	0.65	0.14	8.1
**R**			
-*i*Pr	1.1	0.12	11
*-cyclo*Pentyl	8.2	0.65	17
-C_6_H_5_	1.7	< 0.5	15
-CH_2_OMe	2.3	1.9	80
**(CH_2_)_2_COMe**	**1.6**	**0.15**	**3**
**-OCH_3_**	**1.8**	**0.072**	**1.3**
-OEt	2.9	0.21	6.3
-O-C_6_H_5_	3.5	0.3	24
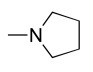	310	>5	>90
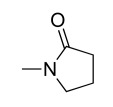	29.5	0.9	12

* Overall biological data were from references [32-33, 38].

**Table 7.** Selected *In Vitro* (IC50, Against *P. falciparum*) and *In Vivo* (ED_50_, Against *P. vinckei*) Antimalarial Activities of Thioester Prodrugs and the Corresponding T3 Drug*

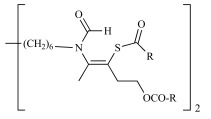	IC_50_ (nM)	ED_50_ (mg/kg) i.p. p.o.	
T3 (parent drug)	2.2	0.2	13
**R**			
-*i*Pr	7	1.2	60
(CH_2_)_2_CO_2_Me	48	0.5	13
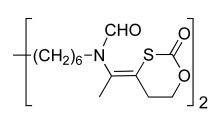	**2.2**	**0.25**	**5**
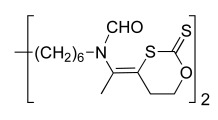	4400		>30

* Overall biological data were from references [32-33, 38].

**Table 8.** Selected *In Vitro* (IC_50_, Against *P. falciparum*) and *In Vivo* (ED_50_, Against *P. vinckei*) Antimalarial Activities of Linker Modified T3 Analogues and the Corresponding T3 Drug*

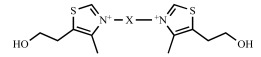	IC_50_ (nM)	ED_50_ (mg/kg) i.p. p.o.	
X =			
(CH_12_)_12_T3 (parent drug)	2.2	0.2	13
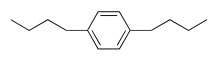	77.5	>> 0.5	>>120
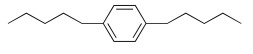	20.5	2.2	53
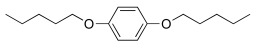	66	> 1	60
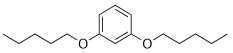	61	>> 5	>> 90
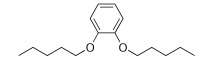	110	>> 5	>> 90

* Overall biological data were from references [39].
